# Chicken lncRNA-9802 Induces the S Phase Arrest in the T Lymphocyte Cells Infected by Marek’s Disease Virus via the TP53BP1/p53/p21 Pathway

**DOI:** 10.3390/vetsci13050469

**Published:** 2026-05-12

**Authors:** Shuo Han, Haile Ren, Jingyi Yang, Kexin Han, Yunqiao Qiu, Yingxue Jiang, Limei Han, Liping Han

**Affiliations:** 1 Engineering Laboratory for Tarim Animal Diseases Diagnosis and Control, College of Animal Science and Technology, Tarim University, Alar 843300, China; 2 Key Laboratory of Livestock Infectious Diseases in Northeast China, Ministry of Education, College of Animal Science and Veterinary Medicine, Shenyang Agricultural University, Shenyang 110866, China; 3 Department of Bioscience, Changchun Normal University, Changchun 130032, China

**Keywords:** Marek’s disease virus, lncRNA-9802, TP53BP1, cell cycle, S phase arrest

## Abstract

The oncogenic Marek’s disease virus (MDV) triggers Marek’s disease (MD), which is a substantial threat to poultry as it transforms infected T cells into tumors. Our research identified that long non-coding RNA 9802 (lncRNA-9802) exhibits increased expression in the chicken spleen following MDV infection, with its expression being strongly associated with the expression of tumor p53-binding protein 1 (*TP53BP1*). The function of lncRNA-9802 in T cells transformed by MDV remains unclear. Consequently, the expression levels of lncRNA-9802 were either over-expressed or knocked down in MDV-transformed T cells, MDCC-MSB1, through lentivirus-mediated over-expression and knock down experiments. Our findings demonstrate that lncRNA-9802 induces proliferation disturbances in MDCC-MSB1 cells by causing arrest in the S phase, which is accompanied by increased expression levels of *TP53BP1*, *p53*, and *p21*. Activation of the p53 pathway results in elevated levels of *Cyclin E* and Cyclin-dependent kinase 2 (*CDK2*), thereby facilitating the entry of MDCC-MSB1 cells into the S phase. Concurrently, the reduced levels of Cyclin A inhibit the exit of MDCC-MSB1 cells from the S phase. By modulating the TP53BP1/p53/p21 pathway, lncRNA-9802 induces S phase arrest in MDCC-MSB1 cells, characterized by upregulation of *Cyclin E* and *CDK2* and downregulation of *Cyclin A*. This research enhances the understanding of the pathogenic mechanisms of MDV and provides a foundation for identifying potential targets for antiviral drug development.

## Simple Summary

Marek’s disease represents a significant viral pathology characterized by tumor formation in chickens, which detrimentally impacts poultry health on a global scale. This study explored the role of lncRNA-9802 in the proliferation of Marek’s disease virus-infected T cells, which exhibits elevated expression levels in the chicken spleen following infection with the Marek’s disease virus. Our findings indicate that increased levels of lncRNA-9802 disrupt the proliferation of these infected T cells by causing cell cycle arrest at the S phase. This arrest is mediated through a signaling cascade involving three key proteins, which subsequently modulate the expression of proteins governing the cell cycle. These findings contribute to the identification of novel therapeutic targets for Marek’s disease, facilitating the advancement of antiviral strategies.

## 1. Introduction

Marek’s disease (MD), triggered by the Marek’s disease virus (MDV), is a highly transmissible disorder involving the abnormal proliferation of lymphoid tissues, endangering the poultry industry on a global scale [1]. MDV is an alpha herpesvirus that causes tumors in T cells and can establish latent infections in the host, ultimately leading to lethal T-cell lymphomas. Even with the extensive use of vaccines, the continuous evolution of viral virulence and the limited protection offered by existing vaccines make MD a persistent global issue, leading to repeated disease outbreaks [2,3]. The financial impact is significant, with annual losses in the United States estimated to be as high as 150 million USD before widespread vaccination was implemented [4]. For this reason, understanding the molecular mechanisms that govern the interaction between MDV and its host is key to developing novel and effective antiviral strategies.

Long non-coding RNAs (lncRNAs) are a type of RNA transcript that is over 200 nucleotides in length and lacks the ability to code for proteins [5]. As regulatory factors, they are vital in gene expression, influencing various levels such as transcriptional, post-transcriptional, and epigenetic regulation [6]. LncRNAs play significant roles in various biological activities, such as cell growth, specialization, programmed cell death, and immune system reactions [7,8,9]. Within viral infections, lncRNAs serve as key regulatory molecules, either contributing to the host’s antiviral strategies or being utilized by viruses to boost their replication and pathogenic effects [10].

Recent investigations have uncovered several lncRNAs associated with MD, such as lincRNA-GALMD3 [11], linc-GALMD1 [12] encoded by the host, and LAT-lncRNA [13] and ERL-lncRNA [14] encoded by MDV. In our former study, chickens were infected with MDV over a span of 42 days, and RNA sequencing was later conducted on the spleen tissues. Our analysis of the lncRNA-mRNA co-expression network in MDV-infected chicken spleens led to the identification of two MD-associated lncRNAs, designated as lncRNA-803 and lncRNA-9802 [15]. It was noted that their expression had a close association with tumor p53-binding protein 1 (*TP53BP1*), pointing to a potential regulatory interaction. Additionally, it was previously demonstrated that lncRNA-9802 operates as a molecular sponge for miR-1646, modulating Bax and Bcl-2 expression and facilitating the proliferation of DF-1 cells [16]. Derived from chicken embryo fibroblasts, DF-1 cells only partially represent the role of lncRNA-9802 in normal chicken somatic cells. The expression of lncRNA-9802 is significantly increased only in response to MDV infection. Performing functional analyses of lncRNA-9802 in MDV-transformed T cells can more effectively investigate the role of lncRNA-9802 in MDV pathogenesis.

As we have described, lncRNA-9802 is strongly correlated with the expression level of *TP53BP1*. *TP53BP1* is an important upstream regulatory factor of *p53*. It has been demonstrated that MDV causes an upregulation of p53 protein expression [15,17]. Research indicates that the viral oncoprotein Meq associates with p53, hindering its transcriptional and apoptosis-inducing roles [18]. The p53 pathway functions as a primary regulatory node for responses to cellular stress. The DNA damage response relies significantly on the mediation of *TP53BP1* [19,20]. The direct interaction between TP53BP1 and p53 is vital for activating p53, thereby impacting decisions regarding cell fate [21]. The TP53BP1/p53/p21 signaling axis has been widely studied for its involvement in cell cycle regulation and tumor suppression [22,23]. These findings collectively indicate that the p53 pathway is involved in the pathogenic processes of MDV, particularly concerning cell cycle regulation.

Tumor cell proliferation is a terrifying event that exacerbates the disease process. Both normal and tumor cells require a complete cell cycle for cell proliferation. Cell cycle involves the participation of some important regulatory proteins, such as Cyclin A, Cyclin E, Cyclin-dependent kinase 2 (CDK2), etc. [24,25]. The varying expression levels of these proteins cause an imbalance in the ratio of different division cycles, which in turn impacts cell proliferation. Tumors are characterized by dysregulated cell cycles, causing genomic instability from unchecked cell cycle advancement [26,27]. Some lncRNAs have been shown to be involved in cell cycle regulation by either directly affecting DNA replication or altering the expression of crucial cell cycle regulatory elements [28,29]. Although progress has been made, we still have a limited grasp of the mechanistic role of lncRNAs in the cell cycle, particularly in the setting of viral carcinogenesis.

To determine if lncRNA-9802 influences the proliferation of MDV-transformed tumor cell via cell cycle regulation, we performed over-expression and knock down studies using lentivirus-mediated methods. Following this, the proliferation of MDCC-MSB1 and changes in cell cycle distribution were assessed. The expression levels of *TP53BP1*, *p53*, and *p21* were also detected. To determine which molecular changes caused alterations in cell cycle distribution, we analyzed the expressions of *Cyclin A*, *Cyclin E*, and *CDK2*. By achieving these goals, the study intended to shed light on the role of lncRNA-9802 in cell cycle dysregulation during MDV infection and to introduce new understanding of MDV pathogenesis.

## 2. Materials and Methods

### 2.1. Ethics Statement

The animal study protocol was approved by the Institutional Animal Care and Use Committee of Tarim University (Approval Code: 2022030906; Approval date: 9 March 2022).

### 2.2. Vector, Cell and Animal

In our lab, we maintain the vectors pET-28a-TP53BP1-breast cancer type 1 C-terminal (BRCT), pGEX-4T-1-TP53BP1(BRCT), pCDH, pGreen, pCDH-lncRNA-9802, pGreen-lncRNA-9802-shRNA, pMD2.G, and psPAX2, as well as MDCC-MSB1 and HEK-293T cells. The original pET-28a, pGEX-4T-1, pCDH, pGreen, pMD2.G, and psPAX2 were purchased from the Miaoling Plasmid Company (P0023, P0001, P0268, P0420, P0262, P0261, Miaoling Plasmid, Wuhan, China). MDCC-MSB1 cells were kindly provided by Professor Man Teng from the Henan Academy of Agricultural Sciences. The HEK-293T cells were purchased from the Procell Company (CL-0005, Procell, Wuhan, China). One-day-old female White Leghorn chicks that are specific-pathogen-free and New Zealand white rabbits were purchased from the Kondebio Company (Kondebio, Qingdao, China). The chicks and New Zealand white rabbits were housed at the experimental animal center.

### 2.3. Production and Preparation of Lentiviruses

The packaging vectors pMD2.G and psPAX2 were used to co-transfect HEK-293T cells with pCDH, pGreen, pCDH-lncRNA-9802, and pGreen-lncRNA-9802-shRNA. For the over-expression negative control (OE NC) lentivirus of lncRNA-9802, the plasmids pMD2.G, psPAX2, and pCDH were co-transfected. To produce the over-expressed (OE) lentivirus of lncRNA-9802, the plasmids pMD2.G, psPAX2, and pCDH-lncRNA-9802 were co-transfected. The knock down negative control (KD NC) lentivirus of lncRNA-9802 was produced by co-transfecting pMD2.G, psPAX2, and pGreen. The knocked down (KD) lentivirus of lncRNA-9802 was generated by co-transfecting pMD2.G, psPAX2, and pGreen-lncRNA-9802-shRNA. Transfection was carried out using the PEI 40K reagent (G1802, Servicebio, Wuhan, China). For routine cultivation, these cells were incubated at 37 °C in a 5% CO_2_ atmosphere for 72 h. Following this, the supernatants with lentiviruses for either over-expressed or knocked down lncRNA-9802 were collected. After gathering the viral suspensions, they were concentrated with the lentivirus concentration kit (G1801, Servicebio, Wuhan, China). The concentration of the viral suspension was measured quantitatively with the lentivirus titer detection kit (G1804, Servicebio, Wuhan, China).

### 2.4. Transient Over-Expression and Knock Down of lncRNA-9802 in MDCC-MSB1 Cells

MDCC-MSB1 cells were seeded at a density of 1 × 10^6^ cells per well in 6-well plates and maintained for 24 h. Next, the medium was then replaced with a fresh RPMI-1640 complete medium. A final concentration of 1 × 10^11^ TU/mL lentivirus suspensions, supplemented with 8 μg/mL polybrene, were incorporated. The cells were cultured for another 72 h to over-express or knock down lncRNA-9802. To assess the infection efficiency of lentiviruses, a systematic procedure was employed. Initially, a bright-field image of MDCC-MSB1 cells is captured. Subsequently, the excitation light settings are adjusted to acquire an image of the same cells exhibiting green fluorescence. This fluorescence is indicative of successful infection, as the plasmid utilized for lentivirus packaging incorporates the green fluorescent protein gene. Five corresponding regions from both images are then selected for analysis. In each region, the number of cells visible in the bright-field image and those exhibiting green fluorescence are enumerated. The infection efficiency is determined by calculating the ratio of the total number of fluorescent cells to the total number of bright-field cells, expressed as a percentage. This independent experiment was replicated three times. As the primary objective is to observe infection efficiency, no statistical significance analysis was conducted.

### 2.5. Flow Cytometry

The cell cycle was examined using the cell cycle detection kit (E-CK-A351, Elbascience, Wuhan, China). MDCC-MSB1 cells infected with lentivirus were first washed three times with phosphate-buffered saline (PBS), with each wash taking 2 min. Following this, the cells were collected in PBS and subjected to centrifugation at 300× *g* for 5 min. A total of 5 × 10^5^ MDCC-MSB1 cells were then washed twice with PBS at −20 °C, followed by centrifugation at 300× *g* for 5 min, and resuspended in 300 μL of PBS solution. The cells then were incubated in anhydrous ethanol at −20 °C for 1 h. Following a 5 min centrifugation at 300× *g*, the cells were placed in 1 mL of PBS and incubated at room temperature for 15 min. Following another centrifugation at 300× *g* for 5 min, 100 μL of RNase A reagent was added, and the mixture was incubated at 37 °C for 30 min. Subsequently, 400 μL of PI reagent was added, and the cells were incubated in the dark for 30 min. The analysis was conducted using a BD FACSAria III flow cytometer (Becton, Dickinson and Company, Franklin, MA, USA).

### 2.6. Induction of Protein Expression

A volume of 100 microliters of *Escherichia coli* BL21(DE3) competent cells (CB105, Tiangen, Beijing, China) was gently combined with 1 ng of either pET-28a-TP53BP1 (BRCT) or pGEX-4T-1-TP53BP1 (BRCT). The mixture was then incubated in an ice-water bath for 30 min. Following this, the cells were subjected to a heat shock by transferring them to a water bath maintained at 42 °C for 1 min, after which they were immediately returned to the ice-water bath for an additional 2 min. Subsequently, the culture volume was increased to 10 milliliters with the addition of growth medium, and the cells were incubated overnight at 37 °C with agitation at 180 rpm. The optical density at 600 nm (OD600) of *Escherichia coli* BL21(DE3) cells, transformed with either pET-28a-TP53BP1(BRCT) or pGEX-4T-1-TP53BP1(BRCT), was standardized to 0.8. Subsequently, Isopropyl β-D-1-thiogalactopyranoside (IPTG) (ST098-1g, Beyotime, Shanghai, China) was introduced into the culture medium to achieve a final concentration of 0.1 mM. The cells were incubated at 25 °C with shaking at 200 rpm for a duration of 14 h to induce the expression of TP53BP1(BRCT). Cultures without IPTG supplementation served as controls. Following incubation, 1 mL of the bacterial culture was subjected to centrifugation at 12,000 rpm for 5 min to pellet the bacterial cells. The pellet was resuspended in an equivalent volume of PBS. Subsequently, proceed with centrifugation and resuspend the bacterial pellet in pre-cooled RIPA lysis buffer (P0013B, Beyotime, Shanghai, China). After ultrasonic disruption, the cell lysate was centrifuged at 12,000 rpm for 30 min to separate the supernatant from the pellet. Sodium dodecyl sulfate–polyacrylamide gel electrophoresis (SDS-PAGE) was then conducted on both the supernatant and pellet. Post electrophoresis, Coomassie Brilliant Blue staining (P0003S, Beyotime, Shanghai, China) and subsequent destaining were performed to visualize the protein banding patterns. Finally, the expression of the recombinant protein was analyzed using a gel imaging instrument (ChemiDoc XRS+, Bio-Rad, Hercules, CA, USA).

### 2.7. Preparation of Polyclonal Antibody

The recombinant protein of TP53BP1(BRCT) was purified using the GST-tag protein purification kit (P2260S, Beyotime, Shanghai, China). The production protocol for TP53BP1(BRCT) antibody involved the following steps: (1) A New Zealand white rabbit was initially injected with 400 μg/kg body weight of purified GST-TP53BP1(BRCT) protein and maintained for a duration of two weeks. (2–4) Subsequently, the rabbit received injections of 400 μg/kg body weight of purified GST-TP53BP1(BRCT) protein, followed by a one-week feeding period. This procedure was repeated three times. (5) Blood was then collected from the ear vein and centrifuged at 3000 rpm for 10 min to isolate rabbit serum containing the GST-TP53BP1(BRCT) antibody.

### 2.8. Enzyme-Linked Immunosorbent Assay (ELISA)

The titer of the GST-TP53BP1(BRCT) antibody was evaluated via the ELISA. A microplate was coated with 1 μg/mL of purified recombinant His-TP53BP1(BRCT) protein and incubated overnight at 4 °C. The GST-TP53BP1(BRCT) antibody was serially diluted from 1:1000 (*v*/*v*) to 1:128,000 (*v*/*v*) and added to the microplate. After a 1 h incubation at 37 °C, horseradish peroxidase-conjugated goat anti-rabbit IgG (D110058, Sangon, Shanghai, China), diluted at 1:5000 (*v*/*v*), was introduced. A 10 min color development was conducted using TMB chromogen solution (P0206, Beyotime, Shanghai, China), and the reaction was terminated with 2 mol/L H_2_SO_4_. Absorbance was measured at 450 nm, and the positive-to-negative (P/N) value was calculated as the ratio of the optical density at 450 nm (OD450) of the positive serum to that of the negative serum for TP53BP1 antibody. A P/N ratio equal to or greater than 2.1 indicates that the antibody at this dilution is considered effective.

### 2.9. Real-Time Quantitative Polymerase Chain Reaction (RT-qPCR)

Wash the MDCC-MSB1 cells, which have been infected with lentivirus for 72 h, with pre-cooled PBS, repeating this process three times. Total RNA from MDCC-MSB1 cells infected with pCDH, pGreen, pCDH-lncRNA-9802, pGreen-lncRNA-9802-shRNA lentiviruses were extracted using RNAiso (DP430, Tiangen, Beijing, China). cDNA was synthesized using HiScript III RT SuperMix for qPCR (+gDNA wiper) (R323, Vazyme, Nanjing, China). The expression levels of genes were quantified using ChamQ SYBR qPCR Master Mix (Q311, Vazyme, Nanjing, China). The primer sequences used for RT-qPCR are listed in Table S1. The relative expression levels of lncRNA-9802 and genes were calculated using the 2^−ΔΔCt^ method. The results are presented as relative fold changes relative to the control group after normalization to the endogenous control GAPDH.

### 2.10. Western Blot

Treat the MDCC-MSB1 cells with pre-cooled RIPA lysis buffer (P0013B, Beyotime, Shanghai, China) and allow lysis to occur at a low temperature for 15 min. Transfer the resulting lysate to a low-temperature centrifuge and centrifuge at 12,000 rpm for 15 min. Collect the protein solution from the supernatant and determine the protein concentration using the BCA protein assay kit. Normalize the protein concentration across samples. Finally, perform the Western blot experiment following standard protocols. Equal amounts of total protein in each lane were transferred to polyvinylidene fluoride membrane. The membranes were blocked using 5% nonfat dried milk for 2 h at 37 °C. The membranes were incubated with His-TP53BP1(BRCT) (1:2000), GST-TP53BP1(BRCT) (1:2000), p53 (1:20,000, Proteintech, Wuhan, China), p21 (1:2000, YM8364, Immunoway, Suzhou, China), CDK2 (1:2000, YM8146, Immunoway, Suzhou, China), Cyclin A (YT1167, 1:2000, Immunoway, Suzhou, China) and GAPDH antibodies (1:150,000, Affinity, China) overnight at 4 °C. Then the membranes were incubated with horseradish peroxidase-conjugated goat anti-rabbit IgG (1:10,000, Sangon, Shanghai, China) for 1 h at 37 °C. The bands were visualized using an enhanced chemiluminescent direct labeling system (P0018S, Beyotime, Shanghai, China). ImageJ software (National Institutes of Health, Bethesda, MD, USA) was employed to quantify the gray value of protein bands.

### 2.11. Statistical Analysis

All experiments were conducted with three independent biological replicates (n = 3), each measured in three technical replicates. Statistical comparisons between two distinct groups (lncRNA-9802 OE NC vs. lncRNA-9802 OE; lncRNA-9802 KD NC vs. lncRNA-9802 KD) were performed using the independent-samples t-test in SPSS 19.0 (IBM, Armonk, NY, USA). Data are presented as mean ± standard deviation, with statistical significance defined as *p* < 0.05 and extreme significance as *p* < 0.01.

## 3. Results

### 3.1. The Effect of lncRNA-9802 Over-Expression and Knock Down on the Proliferation of MDCC-MSB1 Cells

**Figure 1:**
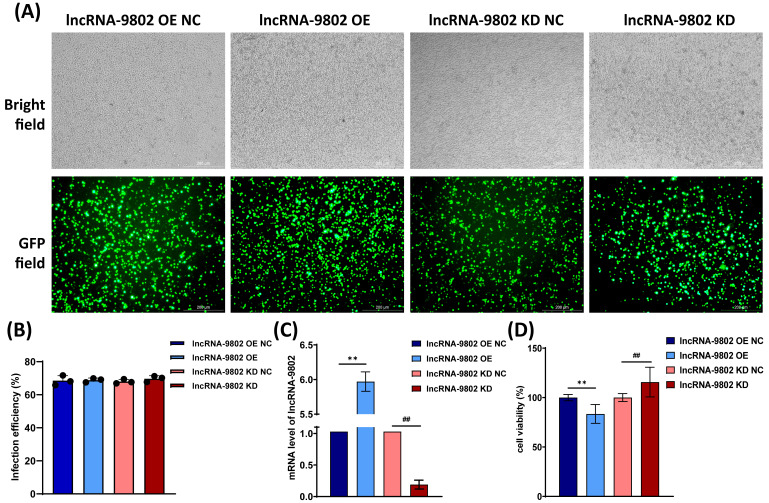
Effect of lncRNA-9802 on the proliferation of MDCC-MSB1 cells assessed by lentivirus-mediated over-expression and knock down. Note: (**A**) MDCC-MSB1 cells were subjected to infection with lentivirus designed for the over-expression (OE) and (KD) of lncRNA-9802 for a duration of 72 h. Successful infection of MDCC-MSB1 cells is indicated by green fluorescence. (**B**) Statistical results of the lentivirus infection efficiency. (**C**) Expression levels of lncRNA-9802 in MDCC-MSB1 cells infected by the lentivirus over-expressing or knocking down for lncRNA-9802. Data are expressed as relative fold changes compared to the negative control (NC) group, following normalization to the endogenous control GAPDH. (**D**) The effect of l ncRNA-9802 on the proliferation of MDCC-MSB1 cells. The independent sample t-test in IBM SPSS Statistics was used to compare differences either between lncRNA-9802 OE NC and lncRNA-9802 OE, or between lncRNA-9802 KD NC and lncRNA-9802 KD. ** represents extremely significant differences between lncRNA-9802 OE NC and lncRNA-9802 OE (*p*  <  0.01). ## indicates extremely significant differences between lncRNA-9802 KD NC and lncRNA-9802 KD (*p*  <  0.01).

MDCC-MSB1 cells are suspension-cultured avian immune cells. Early efforts to use liposome-mediated transfection or electroporation did not succeed in over-expressing or knocking down the expression of lncRNA-9802. Consequently, lentiviruses to over-express and suppress lncRNA-9802 were produced in HEK-293T cells. We noted infection efficiencies greater than 65% in these four groups through fluorescence microscopy following a 72 h infection of MDCC-MSB1 cells, confirming effective lentivirus infection (Figure 1A,B). Then the expression levels of lncRNA-9802 in these cells were detected. The results showed that compared to the over-expression NC or knock down NC, MDCC-MSB1 cells infected with the over-expression or knock down lentivirus showed extremely significant increases or decreases in lncRNA-9802 expression (Figure 1C). It shows the successful development of MDCC-MSB1 cells with altered lncRNA-9802, which can be used for experiments validating the functional deficiency in lncRNA-9802. To further elucidate the role of lncRNA-9802 in the proliferation of MDCC-MSB1 cells, we conducted an analysis of cell proliferation. Subsequent assays revealed that the over-expression of lncRNA-9802 significantly inhibited the proliferation of MDCC-MSB1 cells, and the knock down showed the opposite trend in lncRNA-9802 expression (Figure 1D).

### 3.2. The Effect of lncRNA-9802 on the Cell Cycle Regulation of MDCC-MSB1 Cells

**Figure 2:**
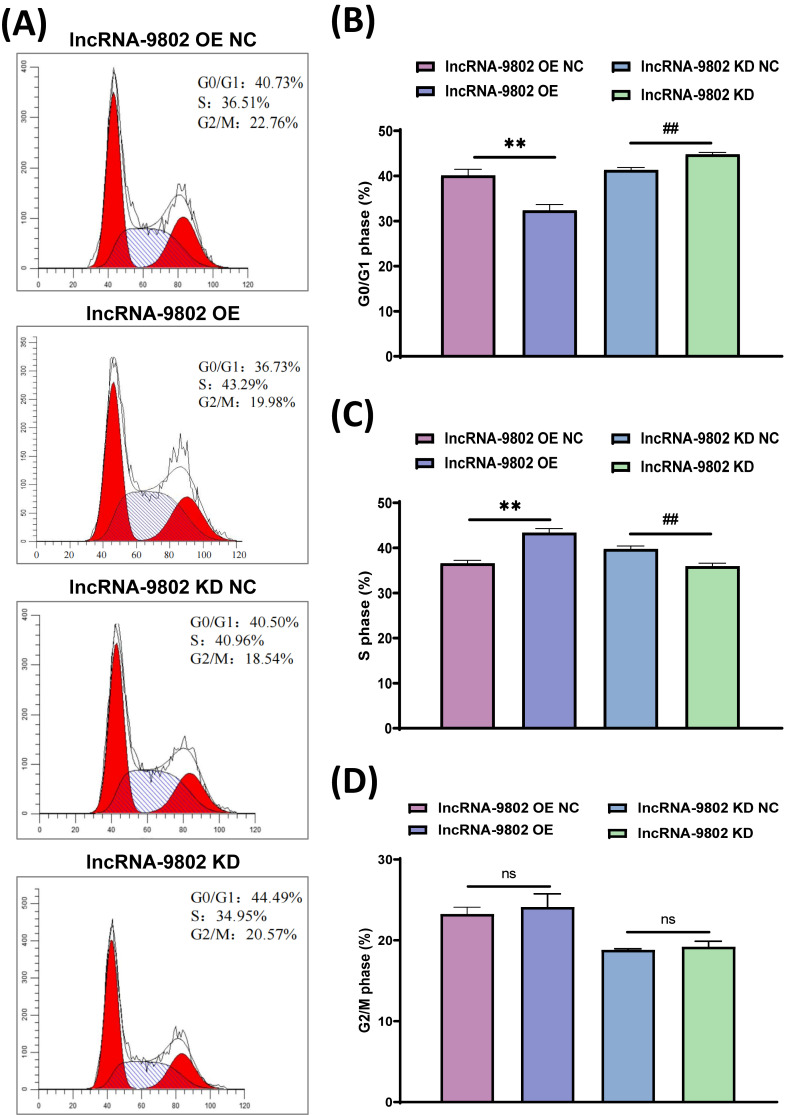
Impact of lncRNA-9802 on cell cycle arrest in MDCC-MSB1 cells. Note: (**A**) Flow cytometry analysis results. The initial red peak along the horizontal axis indicates the proportion of cells in the G0/G1 phase, the subsequent red peak denotes the proportion of cells in the G2/M phase, and the intermediate peak, characterized by stripes, represents the proportion of cells in the S phase. (**B**) The statistical results of the proportion of MDCC-MSB1 cells in the G0/G1 phase. (**C**) The statistical results of the proportion of MDCC-MSB1 cells in the S phase. (**D**) The statistical results of the proportion of MDCC-MSB1 cells in the G2/M phase. ** represents there are extremely significant differences between lncRNA-9802 OE NC and lncRNA-9802 OE (*p*  <  0.01). ## indicates there are extremely significant differences between lncRNA-9802 KD NC and lncRNA-9802 KD (*p*  <  0.01). ns represents no significant difference between lncRNA-9802 OE NC and lncRNA-9802 OE, or between lncRNA-9802 KD NC and lncRNA-9802 KD (*p*  >  0.05).

To investigate how the over-expression and knock down of lncRNA-9802 influence the proliferation of MDCC-MSB1 cells, the distribution of cell cycle phases was examined. Flow cytometry analysis demonstrated that, relative to the over-expression NC, the over-expression of lncRNA-9802 led to an extremely significant reduction in the proportion of MDCC-MSB1 cells in the G0/G1 phase and an extremely significant increase in the proportion of cells in the S phase, while the proportion of cells in the G2/M phase remained unchanged. Conversely, in the lncRNA-9802 knock down group, there was an extremely significant increase in the proportion of MDCC-MSB1 cells in the G0/G1 phase and an extremely significant decrease in the proportion of cells in the S phase, with no significant changes observed in the G2/M phase (Figure 2). These results indicate that the over-expression of lncRNA-9802 promotes the arrest of MDCC-MSB1 cells in the S phase.

### 3.3. Production of TP53BP1 Polyclonal Antibody

**Figure 3:**
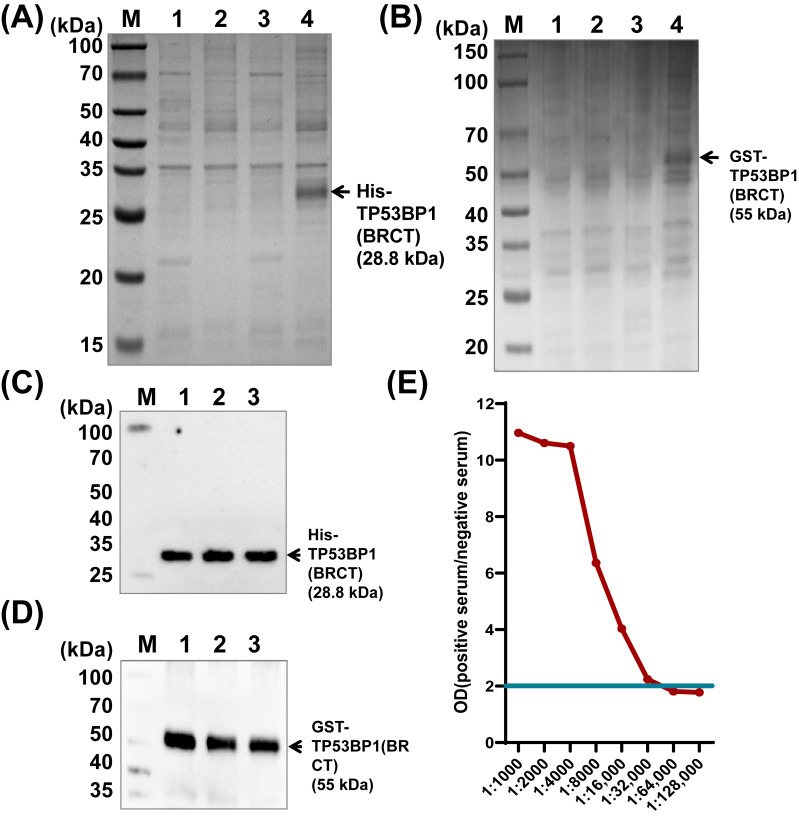
Production of polyclonal antibody against TP53BP1. Note: (**A**,**B**) Expression and purification of recombinant His-TP53BP1(BRCT) and GST-TP53BP1(BRCT) proteins. The M lane represents molecular weight standards ranging from 15 kDa to 150 kDa. Lane 1 corresponds to the supernatant of *Escherichia coli* lysate without IPTG induction. Lane 2 represents the precipitate of *Escherichia coli* lysate without IPTG induction. Lane 3 indicates the supernatant of *Escherichia coli* lysate following induction with IPTG, and lane 4 denotes the precipitate of *Escherichia coli* lysate post-induction with IPTG. (**C**,**D**) The purification of His-TP53BP1(BRCT) (**C**) and GST-TP53BP1(BRCT) (**D**) recombinant proteins was verified via Western blot analysis. The M lane represents molecular weight standards ranging from 15 kDa to 150 kDa, while lanes 1–3 display the purified protein bands of His-TP53BP1(BRCT) and GST-TP53BP1(BRCT) excised from the polyacrylamide gel. (**E**) The titer of the TP53BP1(BRCT) antibody present in rabbit serum. (See Figure S1).

In a previous study, we identified a significant correlation between the expression levels of lncRNA-9802 and TP53BP1, implying that lncRNA-9802 may exert its biological function through the regulation of TP53BP1 expression. To further elucidate the relationship between lncRNA-9802 and TP53BP1 protein expression, a chicken polyclonal antibody specific to TP53BP1 was generated. We constructed the pET-28a-TP53BP1(BRCT) and pGEX-4T-1-TP53BP1(BRCT) expression vectors and subsequently introduced them into *Escherichia coli* BL21(DE3) cells. Induction of recombinant TP53BP1(BRCT) protein expression in BL21(DE3) was achieved using IPTG. Analysis via SDS-PAGE of the total bacterial protein revealed that, after 14 h of induction with 0.1 mM IPTG at 16 °C, there was a notable increase in protein expression at approximately 28.8 kDa (Figure 3A) and 55 kDa (Figure 3B) in both the supernatant and precipitate fractions of the bacterial lysate. The recombinant TP53BP1(BRCT) protein was predominantly localized in the precipitate of the bacterial lysate, suggesting that the applied conditions were effective in inducing the expression of His-tagged and GST-tagged TP53BP1(BRCT) proteins within the bacterial precipitate. Subsequent to the precipitation of samples, we performed protein blotting analysis. The appearance of a singular band corroborated the successful expression of the recombinant His-TP53BP1(BRCT) (Figure 3C) and GST-TP53BP1(BRCT) (Figure 3D) proteins. The recombinant protein was injected into rabbits to generate polyclonal antibody against chicken TP53BP1. Subsequently, the ELISA technique was employed to detect the titer of the TP53BP1 antibody. The results indicated that the calculated P/N ratios surpassed 2.1, corresponding to a serum titer of approximately 1:128,000 (Figure 3E), thus verifying the successful generation of the polyclonal antibody against TP53BP1.

### 3.4. The Effect of lncRNA-9802 on the Expression of the TP53BP1/p53/p21 Pathway in MDCC-MSB1

**Figure 4:**
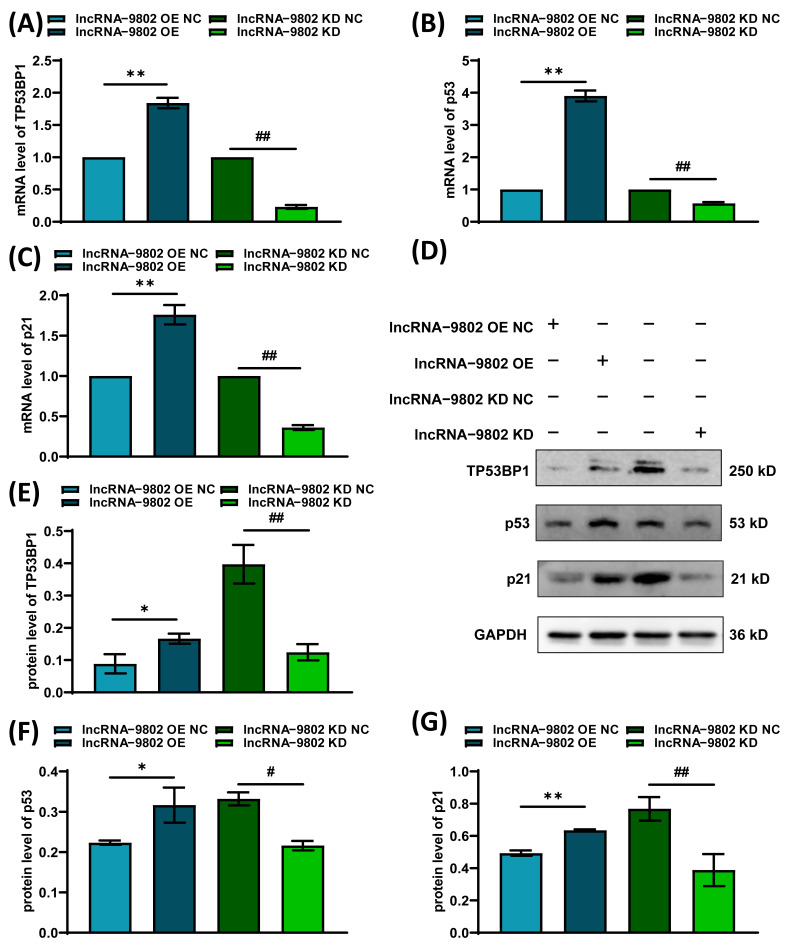
The expression levels of genes associated with the p53 pathway following infection with lentivirus for over-expressing and knocking down lncRNA-9802. Note: (**A**) Relative mRNA expression levels of *TP53BP1*. (**B**) Relative mRNA expression levels of *p53*. (**C**) Relative mRNA expression levels of *p21*. (**D**) Representative Western blot images of the TP53BP1/p53/p21 pathway genes in MDCC-MSB1 cells with over-expressed or knocked down lncRNA-9802. (**E**) Relative protein expression levels of TP53BP1. (**F**) The relative protein expression levels of p53. (**G**) Relative protein expression levels of p21. * represents there are significant differences between lncRNA-9802 OE NC and lncRNA-9802 OE (*p*  <  0.05). ** represents there are extremely significant differences between lncRNA-9802 OE NC and lncRNA-9802 OE (*p*  <  0.01). # indicates there are significant differences between lncRNA-9802 KD NC and lncRNA-9802 KD (*p*  <  0.05). ## indicates there are extremely significant differences between lncRNA-9802 KD NC and lncRNA-9802 KD (*p*  <  0.01). (See Figure S1).

To elucidate the molecular mechanisms through which lncRNA-9802 regulates the cell cycle, the expression levels of *TP53BP1*, *p53*, and *p21* were examined. Our analyses, utilizing RT-qPCR and Western blot techniques, revealed that over-expression of lncRNA-9802 resulted in a significant or extremely significant upregulation of both the mRNA and protein expression levels of *p53*, *TP53BP1*, and *p21*. Conversely, the knock down of lncRNA-9802 led to a significant or extremely significant downregulation of expression levels of *p53*, *TP53BP1*, and *p21* (Figure 4).

### 3.5. The Effect of lncRNA-9802 on the Expression of the Cell Cycle-Related Genes in MDCC-MSB1

**Figure 5:**
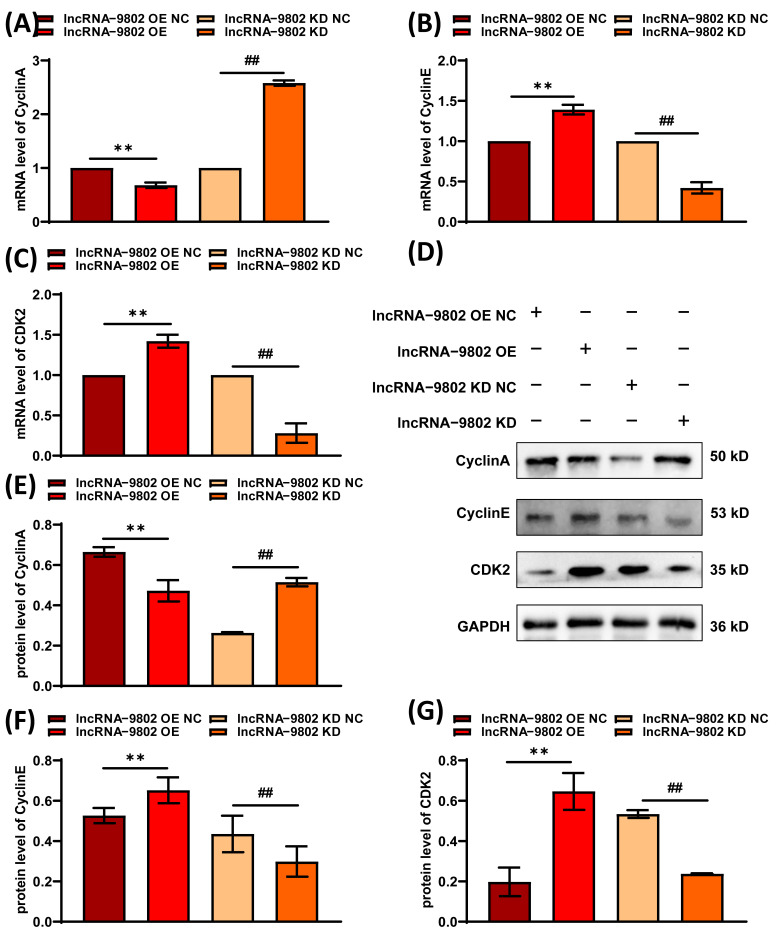
Expression levels of genes related to cell cycle following infection with lncRNA-9802 over-expression and knock down lentiviral vectors. Note: (**A**) The mRNA expression levels of *Cyclin A*. (**B**) The mRNA expression levels of *Cyclin E*. (**C**) The mRNA expression levels of *CDK2*. (**D**) The Western blot results of the key genes in the TP53BP1/p53/p21 pathway. (**E**) The protein expression levels of Cyclin A. (**F**) The protein expression levels of Cyclin E. (**G**) The protein expression levels of CDK2. ** represents there are extremely significant differences between lncRNA-9802 OE NC and lncRNA-9802 OE (*p*  <  0.01). ## indicates there are extremely significant differences between lncRNA-9802 KD NC and lncRNA-9802 KD (*p*  <  0.01). (See Figure S1).

The regulation of the cell cycle is a highly controlled process. Building upon previous findings, our study demonstrates that lncRNA-9802 facilitates the arrest of MDCC-MSB1 cells in the S phase. Nonetheless, the alterations in cell cycle-related protein levels during this process have yet to be elucidated. Consequently, we examined the expression levels of *Cyclin A*, *Cyclin E*, and *CDK2*. Our results indicate that over-expression of lncRNA-9802 leads to a significant increase in the mRNA and protein expression levels of *Cyclin E* and *CDK2*, accompanied by a significant decrease in the expression of *Cyclin A*. Conversely, knock down of lncRNA-9802 results in a significant reduction in *Cyclin E* and *CDK2* expression levels, while *Cyclin A* expression levels significantly increase (Figure 5).

## 4. Discussion

MD is a serious avian disease caused by the oncogenic MDV. MDV infection is characterized by a dynamic progression involving various cell types, with target cells differing at distinct infection stages. Initially, the MDV targets B lymphocytes and macrophages, subsequently shifting its focus to T lymphocytes, where it establishes a latent infection [30]. In chickens that are susceptible to the virus, MDV ultimately induces the transformation of T cells, culminating in the development of T-cell lymphoma [31]. After MDV infection, we previously observed elevated expression of lncRNA-9802 in the chicken spleen [15]. LncRNA-9802 has been shown to enhance the proliferation of the chicken embryo fibroblast cell line DF-1, suggesting its contributory role in the proliferation processes of normal cells [16]. However, the function of lncRNA-9802 in immune cells infected by MDV has not been explored. The MDCC-MSB1 cell line, derived from MD lymphoma, serves as a classical model for MDV research [32]. MDCC-MSB1 harbors the MDV genome but does not produce infectious virus particles and is utilized as an in vitro model for investigating virus-induced T-cell lymphoma [33].

MDCC-MSB1 cells constitute a type of avian immune cell line in suspension culture, which presents significant challenges for transfection [34]. In the preliminary phase of this study, efforts to achieve transfection in MDCC-MSB1 cells via liposome-mediated plasmids were unsuccessful. Consequently, a lentiviral vector system was adopted. This system comprised the encapsulating plasmid pMD2.G, the packaging plasmid psPAX2, and the transfer plasmid such as pCDH-lncRNA-9802 or pGreen-lncRNA-9802-shRNA [35]. Lentiviral vectors are advantageous due to their broad infection spectrum, ability to infect both dividing and non-dividing cells, and capacity for stable integration of exogenous genes into the host genome, making them particularly suitable for transfecting suspended cells [36,37]. In this work, we successfully over-expressed and knocked down the expression of lncRNA-9802, thereby establishing a critical foundation for the comprehensive investigation of the biological functions of lncRNA-9802 in avian immune cells. This outcome not only highlights the resistance of suspended cells to conventional chemical transfection methods but also underscores the importance of selecting appropriate genetic manipulation tools for such cells.

Further investigations were conducted to determine the role of lncRNA-9802 in the proliferation of MDCC-MSB1 cells. In contrast to our findings in DF-1 cells [16], the over-expression of lncRNA-9802 was observed to inhibit the proliferation of MDCC-MSB1 cells. This observation suggests that lncRNA-9802 possesses anti-tumor properties. It is plausible that lncRNA-9802 functions as a host-encoded tumor suppressor or antiviral defense molecule. Given that MDV infection induces the transformation of T cells into tumor cells and that lncRNA-9802 is highly expressed in the chicken spleen, it is likely that this represents an active defense mechanism. The host may upregulate the expression of lncRNA-9802 to inhibit the abnormal proliferation of MDV-infected cells, thereby counteracting the tumor-promoting effects of MDV. The functional disparities of lncRNA-9802 between DF-1 cells and MDCC-MSB1 cells may be attributed to two primary factors. Firstly, the genetic background differences between DF-1 and MDCC-MSB1 cells could account for these variations. DF-1 cells are derived from normal chicken embryos, where fibroblasts undergo spontaneous immortalization during in vitro culture. These cells are characterized by the absence of oncogenic potential, as they do not harbor endogenous genes of common avian viruses and lack numerous oncogenes [38]. In contrast, MDCC-MSB1 cells are derived from spleen tumor tissues of chickens infected with MDV. MDV targets and transforms T lymphocytes, inducing their malignant proliferation, therefore, MDCC-MSB1 cells are classified as a virus-induced tumor cell line. The genome of MDCC-MSB1 cells incorporates the DNA sequence of MDV [39]. Secondly, it is plausible that lncRNA-9802 may produce novel coding peptides, which could interact with the pathogenic proteins of MDV present in MDCC-MSB1 cells, thereby influencing the cellular proliferation. In individuals afflicted with the infection, the virus significantly induces cellular proliferation and transformation of tumor cells [40]. Therefore, we hypothesize that the increased expression of lncRNA-9802 is caused by MDV infection. Upon recognition of this pathological event, the host organism responds by upregulating lncRNA-9802 as a countermeasure to halt this process. Nonetheless, the expression level or duration of action of lncRNA-9802 at the physiological level may be inadequate to fully avert tumor development. Therefore, although we could observe an increase in lncRNA-9802 in the chicken spleen, the MD still remained in a progressing state. In MDCC-MSB1 cells, we artificially strongly upregulated the level of lncRNA-9802. In vitro experiments demonstrate that the exogenous administration of lncRNA-9802 at concentrations substantially exceeding physiological levels effectively suppressed the proliferation of MDCC-MSB1 cells. This finding substantiates the direct inhibitory role of lncRNA-9802 in MDCC-MSB1 cell proliferation.

The cell cycle, essential for cell proliferation, is made up of different phases that are meticulously regulated. The S phase represents a critical juncture in the cell cycle, characterized by DNA replication [41]. Under typical conditions, cells transition from the S phase to the G2 phase, eventually culminating in mitosis (M phase) [42]. However, if DNA replication is obstructed or disrupted, cells are unable to complete the S phase and cannot advance to subsequent stages of division. Further research has demonstrated that the over-expression of lncRNA-9802 induces cell cycle arrest at the S phase in MDCC-MSB1 cells, thereby inhibiting their proliferation. A plausible mechanism underlying this phenomenon is that lncRNA-9802 may elicit DNA damage, thereby activating the DNA damage checkpoint during the S phase and effectively arresting cells to facilitate repair processes [43]. The over-expression of lncRNA-9802 is associated with a decreased proportion of cells in the G0/G1 phase and a significant increase in the proportion of cells in the S phase. This observation indicates an accelerated progression of cells through the G1 checkpoint into the S phase; however, there appears to be an impediment in the transition to the G2 phase, resulting in an accumulation of cells within the S phase.

Upon further examination of gene expression levels in MDCC-MSB1 cells that over-expressed lncRNA-9802, it was observed that the mRNA and protein levels of *TP53BP1*, *p53*, and *p21* were elevated. *TP53BP1* plays a pivotal role in the DNA damage response by rapidly responding to DNA double-strand breaks and being recruited to the damage site to facilitate the selection of appropriate repair pathways [44]. The upregulation of *TP53BP1* provides substantial evidence that DNA damage has occurred within the cell. *p53*, often referred to as the “guardian” of the genome, is typically maintained at low and unstable protein levels under normal conditions. Its marked increase is generally indicative of cellular detection of stress signals, especially those related to DNA damage and replication stress [45]. Upon the over-expression of lncRNA-9802, there is a notable increase in the expression of both *p53* and *TP53BP1*. The protein p53 functions as a crucial mediator of stress response and acts as a tumor suppressor, while TP53BP1 is integral to the DNA damage response pathway [46]. Notably, p21 serves as a critical effector of p53 and functions as a cell cycle-dependent kinase inhibitor [47]. The elevated levels of these proteins imply that the over-expression of lncRNA-9802 may lead to DNA damage or replication stress in MDCC-MSB1 cells.

The cell cycle comprises a series of systematically organized events that facilitate cellular growth and division [48]. In eukaryotic organisms, the cell cycle is segmented into five distinct phases: G0, G1, S, G2, and M phases [49]. The G0 phase is defined by a quiescent state in which cells temporarily cease to divide [50]. During the G1 phase, cells undergo growth and accumulate essential nutrients in preparation for the subsequent S phase, where DNA replication occurs [51]. During the S phase, the DNA content undergoes replication, thereby guaranteeing that each daughter cell inherits a full complement of genetic material following cell division. Upon the completion of DNA replication, cells transition into the G2 phase, during which it undergoes further preparation for the subsequent M phase. The G2 phase, which follows the S phase and precedes the M phase, involves further cellular growth and preparation for mitosis, which takes place during the M phase [52]. These processes are intricately regulated to ensure precise cell development and renewal. The primary molecules governing the cell cycle are Cyclins and *CDK*s [53,54]. We conducted a detailed investigation of cell cycle-related proteins. It was observed that in MDCC-MSB1 cells with over-expression of lncRNA-9802, *Cyclin E* and *CDK2* expression levels were upregulated, whereas that of *Cyclin A* was downregulated. The Cyclin E/CDK2 complex serves as a crucial kinase that facilitates the transition from the G1 phase to the S phase [55]. Persistent over-expression or hyperactivation of Cyclin E/CDK2 results in cells bypassing the requirement for growth signals and checkpoint surveillance, thereby rapidly progressing into the S phase [56]. Consequently, a substantial number of MDCC-MSB1 cells transition from the G1 phase to the S phase, leading to a marked increase in the proportion of S phase cells.

The expression of *Cyclin A* is initiated in the early S phase, where it associates with *CDK2* [57]. Its primary role is to ensure proper DNA replication and facilitate the seamless transition of cells into the G2/M phase, while also being involved in the monitoring of the S/G2 checkpoint [42]. Cyclin A is a crucial protein that enables cells to effectively complete the S phase and advance in the cell cycle [58]. A reduction in Cyclin A results in diminished Cyclin A/CDK2 activity, decreased DNA replication efficiency, stalled replication forks, activation of the S phase checkpoint, and causes retention of cells in the S phase. Conversely, elevated expression of Cyclin E/CDK2 leads to a rapid influx of cells into the S phase. However, insufficient Cyclin A expression causes cells to enter the S phase without successfully progressing to subsequent stages.

The over-expression of lncRNA-9802 significantly upregulates Cyclin E and CDK2, which are essential proteins that facilitate the transition of cells from the G1 phase to the S phase [59]. This upregulation accounts for the observed reduction in the number of cells in the G0/G1 phase, as cells rapidly progressed into the S phase. Despite the sufficient impetus for S phase entry, the expression level of *Cyclin A* is notably diminished. *Cyclin A* is critical for the continuation of the S phase and the transition into the G2/M phase [60,61]. The diminished expression of *Cyclin A* implies that, although DNA replication is initiated, cells are unable to complete the subsequent stages of the S phase, thereby exacerbating the S phase arrest. Simultaneously, the over-expression of lncRNA-9802 results in a marked increase in *p21* levels. As a downstream target of *p53*, *p21* acts as an inhibitor of *CDK2* [62,63]. This situation presents an apparent paradox, as both *CDK2* and its inhibitor, *p21*, were elevated. This phenomenon likely represents a form of negative feedback regulation, wherein p53, upon detecting replication stress induced by lncRNA-9802 over-expression, activates p21 to inhibit CDK2 activity, thereby arresting cell cycle progression. Moreover, our prior investigation in DF-1 cells indicates that lncRNA-9802 targets miR-1646, thereby influencing cell proliferation through the regulation of apoptosis. The observed alterations in the expression levels of p21 and CDK2 may be associated with miRNA-mediated competitive endogenous RNA mechanisms. Further research is required to elucidate the specific molecules involved in this process.

## 5. Conclusions

Based on a comprehensive analysis of our research findings, we conclude that the over-expression of lncRNA-9802 leads to the inhibition of proliferation in MDCC-MSB1 cells by inducing cell cycle arrest at the S phase. The aberrant upregulation of lncRNA-9802 activates the TP53BP1/p53/p21 pathway, facilitating entry into the S phase through the upregulation of Cyclin E/CDK2, while concurrently hindering S phase progression by downregulating Cyclin A. These findings suggest that lncRNA-9802 serves a novel role as a tumor cell cycle regulatory factor associated with MDV, and that the TP53BP1/p53/p21 pathway may represent a potential target for the development of anti-MDV therapeutics.

## Data Availability

The original contributions presented in this study are included in the article/Supplementary Materials. Further inquiries can be directed to the corresponding authors.

## Supplementary Materials

The following supporting information can be downloaded at: https://www.mdpi.com/article/10.3390/vetsci13050469/s1, Figure S1: Primer sequences for RT-qPCR; Table S1 Primer sequences for RT-qPCR.
