# Supplementary material for: Chicken lncRNA-9802 Induces the S Phase Arrest in the T Lymphocyte Cells Infected by Marek’s Disease Virus via the TP53BP1/p53/p21 Pathway
Source: Vet Sci. 2026 May 12;13(5):469. doi: 10.3390/vetsci13050469 (PMC13211383; doi:10.3390/vetsci13050469)
Supplement: Supplementary file 1 [file vetsci-13-00469-s001.zip › vetsci-4292106-supplementary/vetsci-4292106-Figure S1.pdf]

The original data of the figures in the submitted manuscript

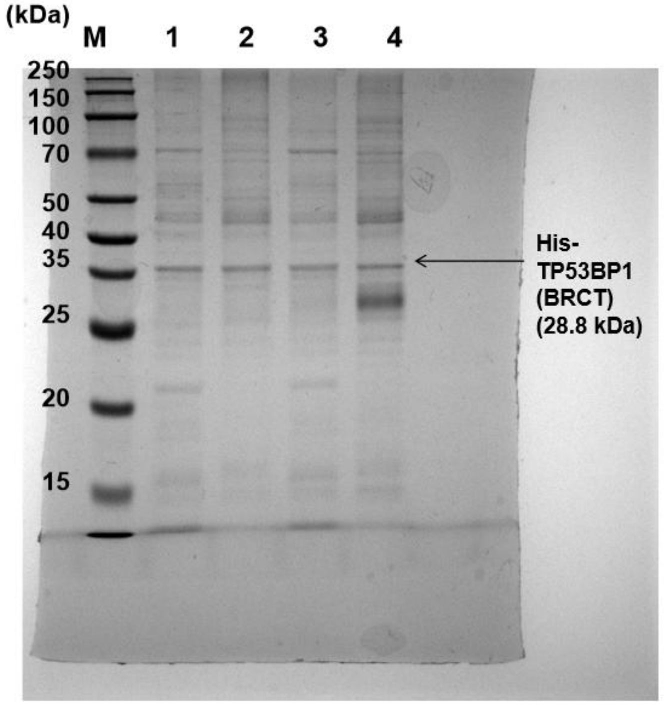

Figure S1. The purification of recombinant His-TP53BP1(BRCT) proteins assessed by SDS-PAGE.
(Original data of Figure 3A in the submitted manuscript)

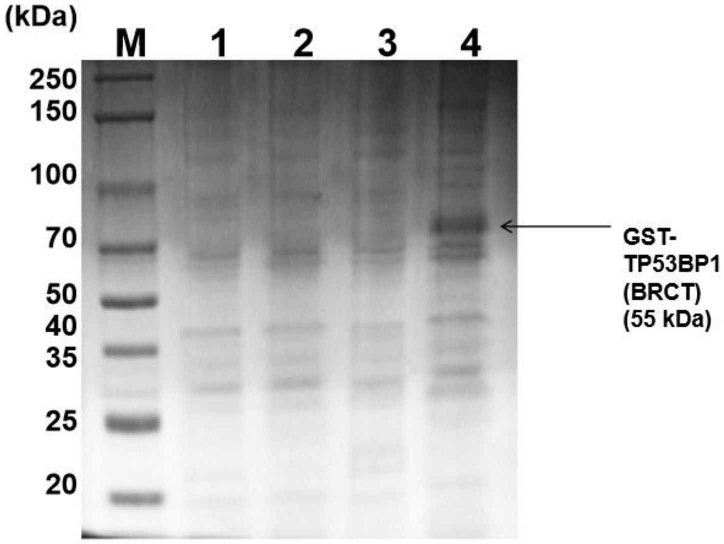

Figure S2. The purification of recombinant GST-TP53BP1(BRCT) proteins assessed by SDS-PAGE.
(Original data of Figure 3B in the submitted manuscript)

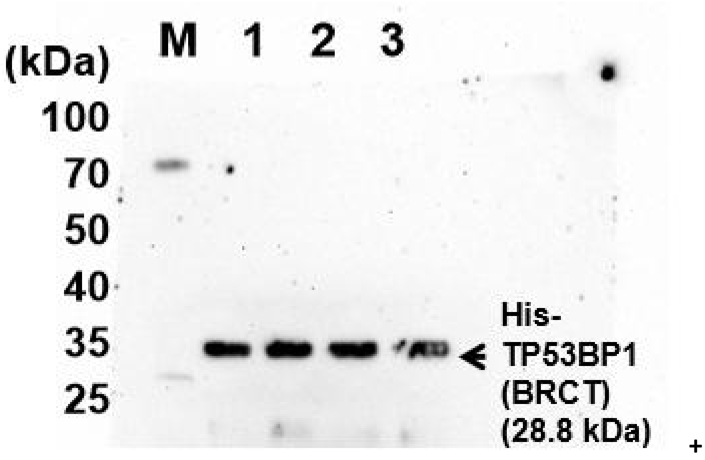

Figure S3. The purification of His-TP53BP1(BRCT) recombinant proteins was verified via Western Blot analysis. (Original data of Figure 3C in the submitted manuscript)

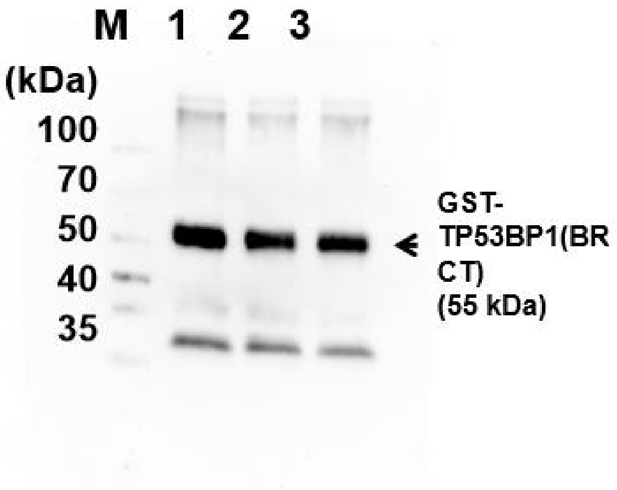

Figure S4. The purification of GST-TP53BP1(BRCT) (D) recombinant proteins was verified via Western Blot analysis. (Original data of Figure 3D in the submitted manuscript)

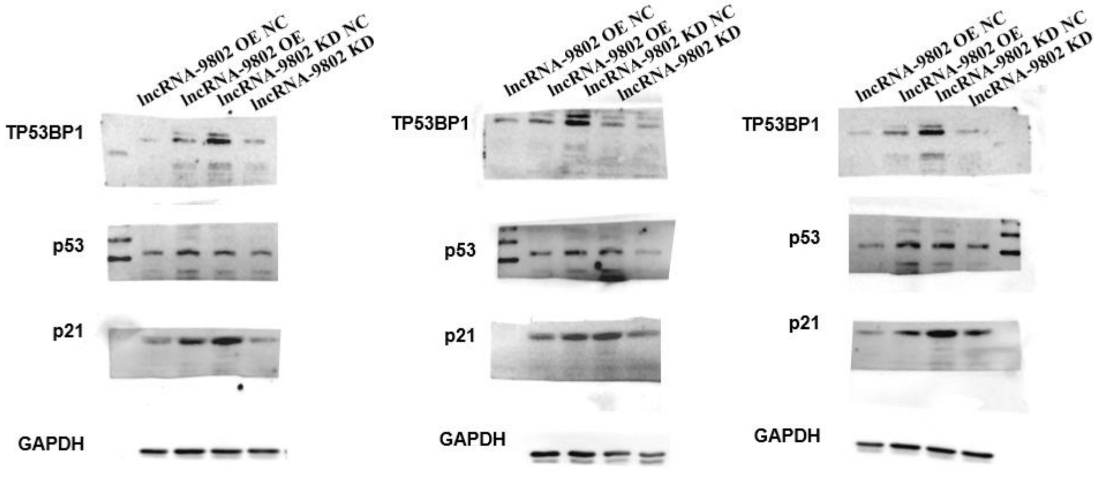

Figure S5. Western Blot results of proteins in the p53 pathway in MDCC-MSB1 cells with overexpression and knockdown of lncRNA-803.
(Original data of Figure 4 in the submitted manuscript)

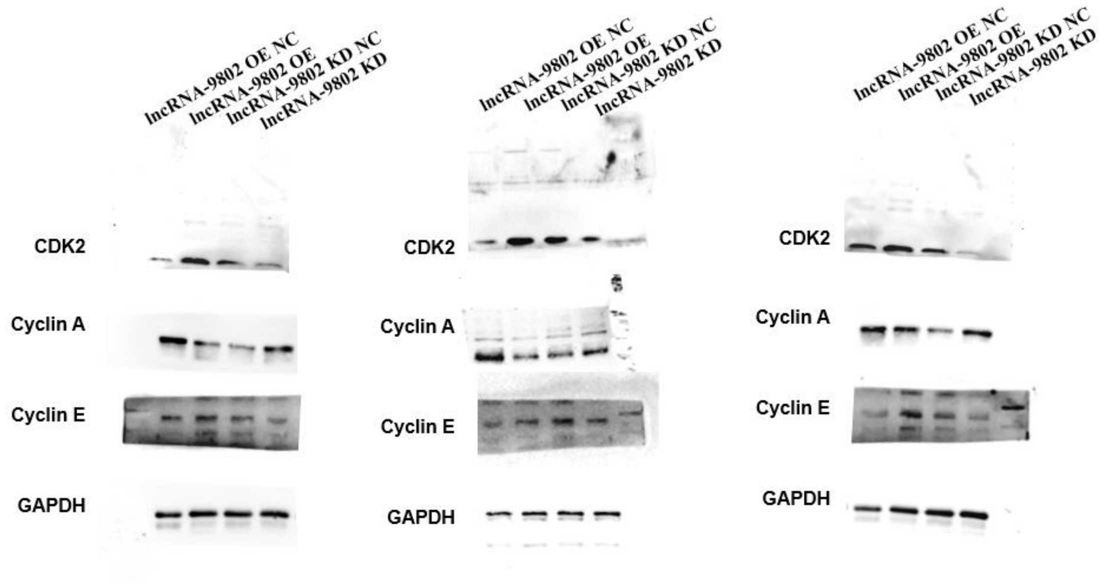

Figure S6. Western Blot results of proteins in the apoptosis pathway in MDCC-MSB1 cells with overexpression and knockdown of lncRNA-803.
(Original data of Figure 5 in the submitted manuscript)
